# Automated Assessment of Movement Impairment in Huntington’s Disease

**DOI:** 10.1109/TNSRE.2018.2868170

**Published:** 2018-09-06

**Authors:** M. Bennasar, Y. A. Hicks, S. P. Clinch, P. Jones, C. Holt, A. Rosser, M. Busse

**Affiliations:** 1 School of Computing and CommunicationsThe Open University 5488 Milton KeynesMK7 6AAU.K.; 2 School of EngineeringCardiff University 2112 CardiffCF24 3AAU.K.; 3 School of BiosciencesCardiff University 2112 CardiffCF10 3AXU.K.; 4 Schools of Biosciences and MedicineCardiff University 2112 CardiffCF10 3AXU.K.; 5 Centre for Trials ResearchCardiff University 2112 CardiffCF14 4YSU.K.; 6 SEWTU, Centre for Trials ResearchCardiff University 2112 CardiffCF10 3ATU.K.

**Keywords:** Accelerometers, upper-limb assessment, Huntington’s disease, movement disorder

## Abstract

Quantitative assessment of movement impairment in Huntington’s disease (HD) is essential to monitoring of disease progression. This paper aimed to develop and validate a novel low cost, objective automated system for the evaluation of upper limb movement impairment in HD in order to eliminate the inconsistency of the assessor and offer a more sensitive, continuous assessment scale. Patients with genetically confirmed HD and healthy controls were recruited to this observational study. Demographic data, including age (years), gender, and unified HD rating scale total motor score (UHDRS-TMS), were recorded. For the purposes of this paper, a modified upper limb motor impairment score (mULMS) was generated from the UHDRS-TMS. All participants completed a brief, standardized clinical assessment of upper limb dexterity while wearing a tri-axial accelerometer on each wrist and on the sternum. The captured acceleration data were used to develop an automatic classification system for discriminating between healthy and HD participants and to automatically generate a continuous movement impairment score (MIS) that reflected the degree of the movement impairment. Data from 48 healthy and 44 HD participants was used to validate the developed system, which achieved 98.78% accuracy in discriminating between healthy and HD participants. The Pearson correlation coefficient between the automatic MIS and the clinician rated mULMS was 0.77 with a p-value < 0.01. The approach presented in this paper demonstrates the possibility of an automated objective, consistent, and sensitive assessment of the HD movement impairment.

## I. Introduction

Huntington’s disease (HD) is an autosomal dominant, progressive neurodegenerative genetic disorder, which affects 11.2 to 13.5 people per 100,000 of the general population. HD is characterised by the development of progressive motor impairment, cognitive decline and behavioural problems [1], [2], caused by an expanded trinucleotide CAG sequence in the Huntingtin (HTT) gene [2]–[4].

One of the most prominent motor symptoms in HD is chorea, which is used to describe abnormal involuntary movement characterized by abrupt, irregular, unpredictable, non-stereotyped movements, However, other motor abnormalities such as dyskinesia, dystonia, rigidity, and bradykinesia are also seen. A critical problem for the evaluation of novel therapeutics is the acknowledged lack of objective clinical measures suitable for evaluating the components of the movement disorder. The Unified Huntington’s Disease Rating Scale (UHDRS) [5] is currently the gold standard to assess disease symptoms in HD. However, UHDRS assessment is limited by inter- and intra-rater variability, subjective bias, and categorical design. Furthermore, the UHDRS score does not relate motor impairment to function in daily life, which is desirable in HD assessment [6].

Over the past twenty years there has been significant progression in human motion recording and analysis over a wide range of applications, including orthopaedic and neurological rehabilitation. Such analysis requires highly accurate motion tracking made possible using, for example, camera-based systems (e.g. Qualisys, Sweden and Vicon, UK). Unfortunately such systems are expensive, difficult to transport, and require dedicated laboratory space, although low-cost but less accurate motion capture devices such as Kinect are also becoming popular in motion analysis research [7].

More recently, there have been a number of attempts to evaluate chorea, dystonia and bradykinesia in people with movement disorders using Inertial Measurement Units (IMUs) or electromagnetic motion sensors [6], [8]–[13]. The majority have relied on statistical methods to assess movement impairment [10], [11], however more recently machine-learning techniques are being applied to the data to achieve automated assessment of the presence or absence of the symptoms or their severity [9], [13]–[15]. Application of machine learning methods for wearable sensor data in Parkinson’s disease (PD) was advocated and explained in detail in a recent review where the possibility of using wearable sensor data for clinical PD measurement was also highlighted [16]. Specifically, the main stages in applying machine learning techniques to the analysis of the sensor data were identified as *feature extraction,* which summarises sensor data into a small set of *features*; *dimensionality reduction*, which further reduces the number of features for ease of analysis and to retain only the most significant information; and *supervised* or *unsupervised learning* which finds patterns in the sensor data. In particular, *supervised learning* learns a model (relationship) between inputs (a set of feature values) and outputs (for example, symptom severity classification) from a set of examples, and uses this model to predict outputs given a new set of input values. At the same time, common pitfalls of machine learning such as *overfitting* and *underfitting* a model were noted along with possible remedies, such as model *complexity control* through the use of, for example, *cross-validation* model testing.

There has been a fair amount of research in the area of applying machine learning methods for the automatic assessment of the movement disorders associated with PD, particularly tremor and bradykinesia [14], [15], [17]–[19]. At the same time, there were only two reports on the application of these methods in HD [9], [13]. In the two latter studies, machine learning techniques were used to automatically classify people into HD and healthy controls groups based on gait analysis [9] or arm movements [13] and data from IMUs. However, these studies are still falling short of proposing an automatic system capable of assessing HD movement impairment using a more sensitive, continuous scale necessary for monitoring the disease progression.

The aim of this study was to apply signal processing and machine learning techniques in the development and validation of a low cost, objective automated system for the evaluation of upper limb movement impairment in HD. Data from 48 healthy and 44 manifest stage HD participants were collected and used to design and validate the proposed system. Signal processing techniques were used to extract time and frequency domain features from the acceleration signals; a feature selection method was used to determine the features important for distinguishing HD patients from healthy controls. The selected features were subsequently used in the classification and quantitative assessment tasks. An ensemble classifier was proposed to distinguish between healthy individuals and those with a diagnosis of HD, which significantly improved the accuracy of the previously proposed simple SVM classifier [13]. Linear regression model was created to generate continuous scale sensitive assessment of movement impairment in HD, which has not been attempted in previous research.

In this research, we limited our focus to the upper limbs during the performance of a functional task both to minimise error in placement of accelerometers during movement and to ensure ease of clinical application of the automated assessment in the future.

## II. Materials and Methods

### A. Participants and Setting

Participants with manifest HD and healthy controls were recruited to this observational study. All participants were provided with a written information sheet describing the research, and their consent was obtained before any data collection. HD participants were eligible if they had a genetically confirmed diagnosis of HD with score of four on the motor diagnostic confidence scale of the UHDRS, were over 18 years of age and recruited onto Enroll-HD, which is a global observational study that provides researchers with access to non-identifiable clinical information (https://www.enroll-hd.org/). They were not eligible if they were unable to provide informed consent. Ethical approval was granted for this study by the South East Wales Research Ethics committee (REC reference: 14/WA/1195) and Cardiff University School of Engineering.

### B. Assessments

Demographic data including age (years), gender and UHDRS-TMS [5] were recorded from the most recent annual Enroll-HD assessment. The UHDRS-TMS provides a clinician observed rating of oculomotor function, dysarthria, chorea, dystonia, rigidity, bradykinesia, balance and gait. Each item was rated by a rater certified clinician on a scale of 0–4, where 0 is equivalent to no impairment. The maximum possible score of all items is 124 (indicating maximum disability). For the purposes of this study a *modified upper limb motor impairment score (mULMS)* was generated from the UHDRS-TMS as the sum of items assessing left and right upper limb dystonia, trunk chorea, and left and right upper limb chorea. Thus mULMS can vary between zero for no impairment to a maximum of 20 representing severe motor impairment.

**Fig. 1.**
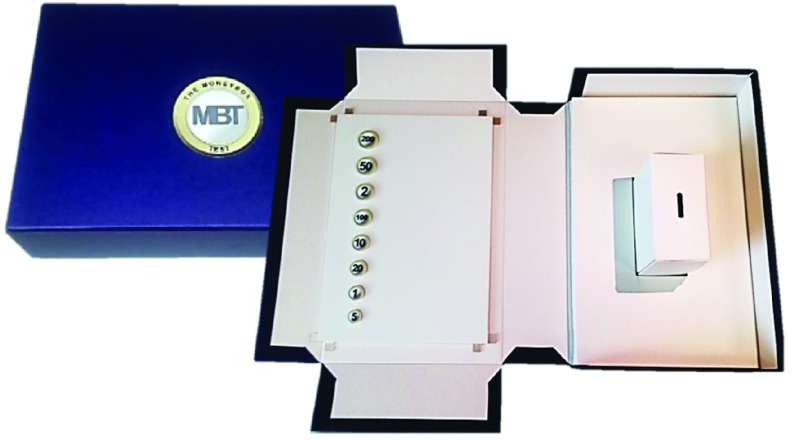
The MBT test enclosed in the case when not in use (left) and open ready for testing (right).

All participants completed the Money Box Test (MBT) [13], [20] - a functional upper limb dexterity assessment involving a series of token transfer tasks (Fig. 1). The tasks increase in difficulty (baseline simple, baseline complex and a dual task). In the baseline simple task, eight blank tokens of varying size are presented and positioned in designated slots, vertically, in size order. The participant is asked to pick up each token individually with their non-dominant hand, transfer it to their dominant hand and place into the moneybox, starting with the largest token and finishing with the smallest. For the baseline complex task, a different set of tokens, with one of the eight values printed on them (1, 2, 5, 10, 20, 50, 100, and 200) is used. In this task, the tokens are positioned in order of size. The participant is asked to transfer the tokens into the moneybox in decreasing value order. The dual task consisted of the same test procedure as the baseline complex, with the participants additionally asked to recite the alphabet simultaneously while transferring the tokens to the moneybox.

### C. Accelerometers

**Fig. 2.**
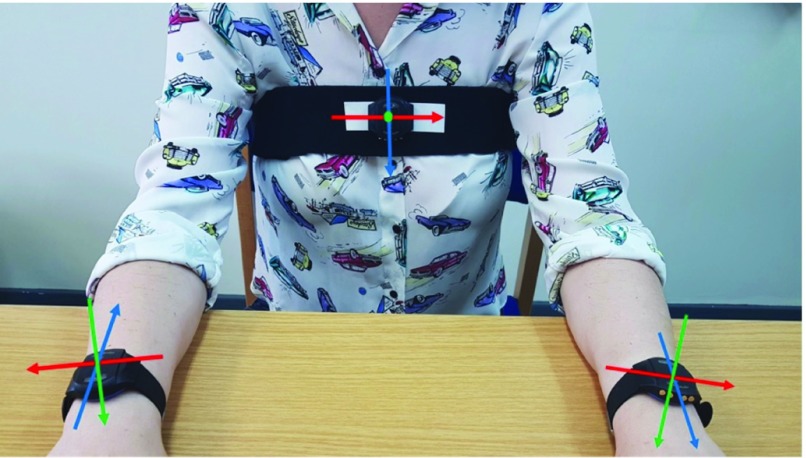
The placement and orientation of accelerometers on the wrists and chest of a participant. In the image, the x-axis is red, y-axis is blue and z-axis is green. The z-axis for the chest sensor (green) is pointing away from the viewer.

Three triaxial GENEactiv accelerometers (Activinsights, UK) were placed on the participant during the performance of the MBT, one on each wrist to record the acceleration of the hands, and one on the chest to capture the movement of the trunk (Fig. 2). Each GENEactiv sensor incorporates three accelerometers, where the accelerometers are orthogonally aligned to each other. The technical specifications of the accelerometers are as following: unit mass 16g, unit size $43\text {mm} \times 40\text {mm} \times 13$mm, sample frequency up to 100Hz, acceleration range ±8g, where g = 9.81 m/s^2^. Before the accelerometers were fixed on the participant, their time settings were synchronized with those of the computer using GENEactive PC software application. The accelerometers did not require any additional calibration. All data were recorded at frequency of 100Hz.

### D. Automatic Classification System

An early version of the system proposed in this article was presented in [13]. In comparison to the full system presented in this article, the system described in [13] was based on a simple SVM classifier and temporal features only and was used to distinguish between HD patients and healthy controls.

**Fig. 3.**
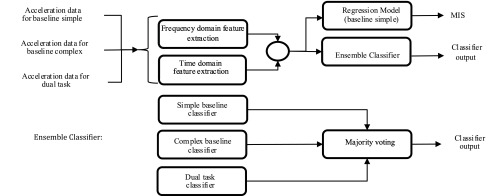
Above: classification system; below: ensemble classifier.

The fully developed system presented in this article consists of three main modules: signal processing and feature extraction including both temporal and frequency domain features, ensemble classifier to distinguish between HD patients and healthy controls during the performance of three different MBT tasks, and a linear regression model to generate continuous scale sensitive assessment of movement impairment in HD (Fig. 3). In this study, both ensemble classifier and the linear regression model are implemented using *supervised learning* techniques.

### E. Signal Processing

**Fig. 4.**
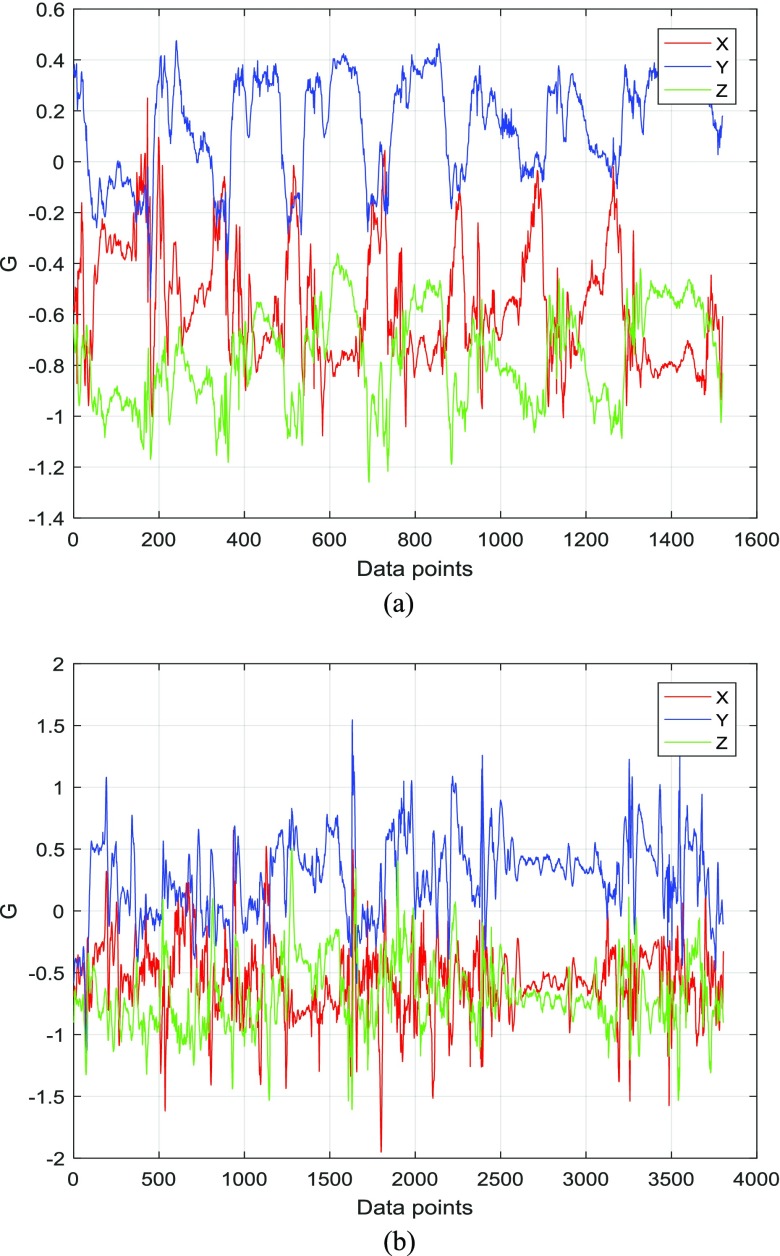
Examples of accelerometer data for the non-dominant hand for: (a) healthy control participant; (b) manifest stage HD patient.

In this part of the system, a range of signal processing techniques are applied to the accelerometer data in order to extract informative features, which potentially could be used for continuous quantification of the movement impairment typical of HD, such as chorea and dystonia, i.e. the abruptness and irregularity of movement and twisting body movements. Each of three MBT tasks consists of eight repeated sub-tasks of transferring a coin from its position to the moneybox, which results in eight observable cycles in motion acceleration data for a healthy person, but not for HD patients, whose motion is characterized by jerky, sudden movements (Fig. 4) [20]. Time and frequency domain features measuring the degree of repeatability, regularity, and recurrence are extracted from the accelerometer data as explained in detail below.

### F. Time Domain Features

This set of features (Table I) includes several features derived directly from the raw accelerometer signals without any filtration or down sampling to ensure that no important information related to the movement impairment is lost.

**TABLE I:** Time Domain Features

Feature	No. of attributes per subject	Feature description	
Recurrence rate	9	Recurrence rate in the signal, i.e. the probability that any state will recur again	RQA
R_Entropy	9	Measurement of the recurrence structure complexity	
Determinism	9	The ratio of recurrence points	
Average diagonal line	9	Average time that signal segments remain the same.	
LE	9	Lypaunov exponent, measures the level of chaos in the time series signal	
Sample entropy	9	Assesses the complexity of the time series signal	
Permutation entropy	9	Assesses the complexity of the time series signal	
Standard deviation	9	The standard deviation of the time series signal	
Mean	9	The mean of acceleration signal	
Correlation between axes	9	Correlation between each pair of axes for each accelerometer	

These features include simple time domain features such as signal mean and standard deviation as well as correlation between the acceleration signals along different axes. Other time domain features used in this study are derived using Recurrence Quantification Analysis of Nonlinear Dynamical Systems (RQA) [21], which quantifies the recurrences of a dynamical system. The values of RQA are expected to be higher for HD than for healthy participants. In addition, Lyapunov exponent (LE) [22] is used to measure the degree of chaos in the signal. As the acceleration signals are less regular and more chaotic for HD patients, a significant difference between the LE values can be expected for these two groups. Sample entropy is used to assess the complexity and regularity within the time series data, as it measures the degree of dependency of a given data point on a number of previous data points [23]. Finally, permutation entropy [24] is used to measure the regularity in the time series data by measuring the existence or absence of permutation patterns within it.

### G. Frequency Domain Features

Frequency domain features are expected to change depending on the presence of involuntary jerky movements in the acceleration data, so short-time Fourier Transform (STFT) is employed to transform the acceleration data to frequency domain. There are eight clearly observable cycles in the acceleration data for a healthy person resulting from the activity of transferring eight coins (Fig. 4). On average, for healthy volunteers, each of the transfer cycles lasts 2 seconds. Therefore, a decision was made to use a sliding window of 2 seconds length and 50% overlap for STFT. In this study, a simple rectangular window followed by a low-pass filter was used. As explained below, only the first five low frequency components are to be used in further calculations, and thus applying a smoothing window such as Hanning was deemed unnecessary.

Three sets of frequency features are extracted from the result of STFT, namely, spectral energy, component entropy, and the average magnitudes of each of the first five STFT components over all windows in each of the tests. These features were chosen as they had been reported to provide good results for movement recognition on the basis of accelerometer signals and thus potentially could contain information useful for measuring the degree of movement impairment in HD patients [25]. Wavelet Packet Decomposition (WPD) is also used to extract a number of time-frequency features from the signals. The decision to employ WPD was made on the basis of its ability to describe signals containing numerous frequency changes over time [26]. In this study, the accelerometer signals are decomposed into five levels using Daubechies 2 wavelets. The six wavelet features used in this study are defined as the sum of the absolute values of coefficients at levels 1–5; these features were chosen as they have been reported to have good ability to capture the patterns of the low frequency movements in the accelerometer signals, which should be suitable for HD data [24]. Table II shows a summary of the extracted frequency domain features.

**TABLE II:** Frequency Domain Features

Feature	No. of attributes per subject	Description of the feature
Frequency domain entropy	9	Entropy of STFT components
Spectral energy	9	Sum of STFT coefficients
Average magnitude of 5 SFFT	45	The average magnitude of the first 5 STFT components for each axis
Magnitude coefficients of wavelet	63	The sum of absolute values of wavelet coefficients at levels 1–5
Wavelet energy	9	Sum of squared wavelet coefficients
Wavelet entropy	9	Entropy of wavelet component

### H. Feature Selection

Following the initial feature extraction, when a total of 234 time and frequency domain feature values are extracted from the signals of the three sensors for each participant and each MBT task, a feature selection method is used to select the most relevant and non-redundant features for discriminating between HD and healthy participants and thus to reduce the dimensionality of the data set. In this study the Joint Mutual Information Maximisation (JMIM) [27] feature selection method is chosen as it relies on an objective function to select the most informative features from a set and has been reported to outperform the other state of the art methods. Continuous features are discretized using Equal Width Discretization method (EWD) [28] before JMIM is applied to all features.

### I. Ensemble Classifier

The goal of this part of the system is to distinguish between HD patients and healthy participants, for which an ensemble classifier is created. A *supervised learning* approach is followed to create an ensemble classifier, the inputs for which are the values of the selected features, and the output takes on one of the two values: HD or healthy control. To make the classifier more sensitive to the detection of HD patients at the early manifest stage of HD, the data from all three MBT tasks (if available) is used for all HD and healthy participants. For each MBT task, a Support Vector Machine (SVM) classifier with radial basis function (RBF) kernel [29] is trained using a number of the most significant features extracted from the corresponding dataset (Fig. 3). Thus the first classifier is trained on the selected features for the baseline simple dataset, the second classifier is trained on the selected features for the baseline complex dataset and the third classifier is trained on the selected features for the dual task dataset. Finally, the results of the three classifiers are combined in an ensemble classifier. Different techniques for combining results of different classifiers such as Bayes, majority voting, and decision template [30] were tested, from which the majority voting was chosen as providing the best results. In some cases, when the participant failed to perform the baseline complex or the dual task (due to the advanced stage of the disease), the classification was performed using only the baseline simple classifier.

### J. Linear Regression Model

In this stage, a linear regression model is used to automatically generate the Movement Impairment Score (MIS) intended to describe the degree of impairment related to the upper limb movement. Five of the most significant features extracted from the baseline task MBT dataset are used as independent variables (inputs) and the mULMS is used as a dependent variable (output). SVM linear regression is applied to obtain the regression model parameters and leave-one-out cross-validation is employed to assess the correlation between the MIS and the mULMS.

## III. Results

Mean (SD) age in years in HD participants (n = 44, 26 males) and healthy controls (n = 48, 26 males) were 53.49 (13.19) and 37.38 (13.31) respectively. Mean (SD) scores on the UHDRS-TMS and mULMS for HD participants were 36.43(23.16) and 5.98 (4.17) respectively. Seven HD participants failed to perform both the baseline complex and the dual tasks, while five other HD participants failed to perform the dual task only. The average time taken to perform the test by the HD participants was 30.62 seconds. The average time for the healthy controls was 13.6 seconds.

### A. Significant Features

The most significant features for discrimination between HD and healthy participants were identified using the JMIM method for the baseline simple, baseline complex, and dual tasks (Tables III–V). The results showed that the recurrence rate feature of the non-dominant hand X-axis was significant for discriminating between HD patients and healthy controls in all three MBT tasks. This can be explained by the fact that most of the transfer task was performed by the non-dominant hand with the dominant hand remaining close to the moneybox. The results also showed that time domain features measuring regularity, repeatability, and chaos were more significant than the frequency domain features, which means that time domain features represent the movement patterns specific to chorea and dystonia better than other features.

**TABLE III:** Ten Most Significant Features for Discriminating Between HD and Healthy Controls in the Baseline Simple Task

No	Feature
1	Non-dominant hand X-axis recurrence rate (RQA)
2	Non-dominant hand Y-axis standard deviation
3	Non-dominant hand Y-axis recurrence entropy (RQA)
4	Chest X-axis sample entropy
5	Dominant hand Z-axis sample entropy
6	Dominant hand Y-axis mean
7	Dominant hand Y-axis sample entropy
8	Dominant hand Y-axis wavelet entropy
9	Chest correlation between X axis and Z axis
10	Chest Z-axis sample entropy

**TABLE IV:** Ten Most Significant Features for Discriminating Between HD and Healthy Controls in the Baseline Complex Task

No	Feature
1	Non-dominant hand X-axis recurrence rate
2	Dominant hand Z-axis sample entropy
3	Dominant hand Y-axis average diagonal line (RQA)
4	Dominant hand X-axis CC5 magnitude coefficients of wavelet decomposition
5	Dominant hand Y-axis sample entropy
6	Dominant hand X-axis Lypaunov exponents
7	Chest Y-axis sample entropy
8	Dominant hand Z-axis permutation entropy
9	Dominant hand X-axis average diagonal line (RQA)
10	Chest Z-axis mean

**TABLE V:** Ten Most Significant Features for Discriminating Between HD and Healthy Controls in the Dual Task

No	Feature
1	Non-dominant hand X-axis recurrence rate (RQA)
2	Non-dominant hand Y-axis permutation entropy
3	Non-dominant hand X-axis average diagonal Line (RQA)
4	Dominant hand Y-axis sample entropy
5	Dominant hand correlation between Y axis and Z axis.
6	Non-dominant hand Z-axis mean
7	Non-dominant hand Y-axis determinism (RQA)
8	Chest X-axis recurrence entropy
9	Chest Z-axis recurrence rate
10	Chest Y-axis recurrence rate

### B. Performance of Ensemble Classifier

The performance of each classifier for discriminating between healthy controls and HD patients was tested separately as well as in combination as an ensemble classifier. The ranking of features provided by the feature selection method was used to find the best subset of features for each classifier. Five folds cross-validation was used to train and test the classifiers. This means that the data related to different participants was divided randomly into five folds (sets of approximately equal size), with four folds used for training and the remaining fold used for testing. The cross-validation process was repeated five times until each fold was used for testing exactly once. Thus in each iteration the training and testing data were different. The average accuracy, sensitivity, and specificity over five iterations were used as the measure of the classification performance.

In the experiment, the first classifier was trained and tested using the baseline simple MBT dataset. For this classifier, a combination of 43 most significant features produced the best performance with accuracy of 91.11%, sensitivity of 90.38%, and specificity of 92.11%. The second classifier was trained and tested using the baseline complex MBT dataset, for which the best performance was achieved using only three features, with the classification accuracy in discriminating between HD patients and healthy controls of 93.1%, and sensitivity and specificity of 87.76% and 100% respectively. Finally, the third classifier was trained and tested using the dual task MBT dataset. The best accuracy of 87.8% was achieved using 49 most significant features. The corresponding values for sensitivity and specificity were 81.82%, and 94.74% respectively.

**Fig. 5.**
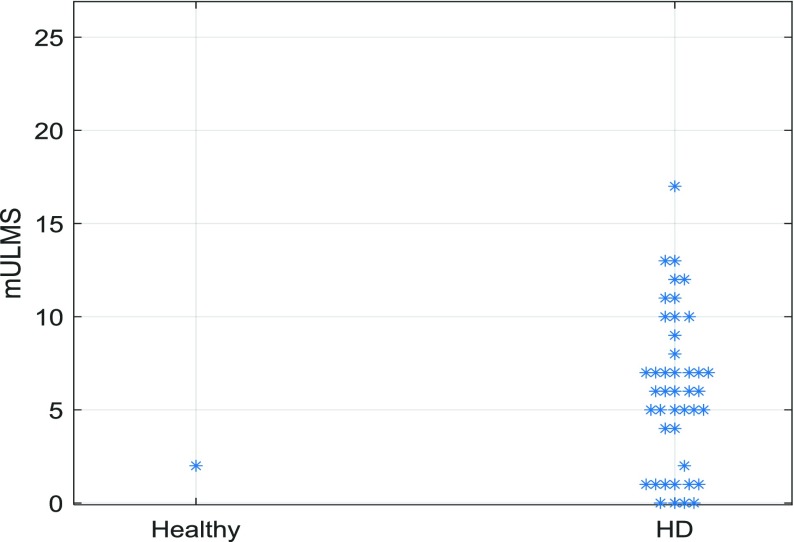
The output of the ensemble classifier vs the mULMS for HD patients.

The ensemble classifier obtained by majority voting between the above three classifiers achieved the accuracy of 98.8%, 100% specificity and 97.7% sensitivity with only one out of 44 HD participants misclassified as a healthy control, and all healthy controls classified correctly. This compares well to the accuracy of 86.4% reported in [13] in a similar experiment. The misclassified HD case had a very low mULMS equal to 2, indicative of very subtle motor symptoms, which can explain the error, although 11 more patients with the mULMS of 2 or lower were classified correctly as HD patients (Fig. 5).

Considering that the features related to the chest sensor often appeared at the bottom of Tables III–V, it was decided to evaluate the performance of the ensemble classifier without the features related to the chest sensor in order to test the hypothesis that the chest sensor was not needed to achieve accurate classification of HD and healthy control participants.

In this experiment, the best accuracy for the baseline simple MBT classifier was achieved using 60 most significant features. For the baseline complex MBT classifier, the best accuracy was achieved using 49 most significant features, and the best accuracy for the dual task MBT classifier was achieved using 25 most significant features. The achieved accuracy, sensitivity and specificity for the ensemble classifier were 86.52%, 83.78% and 88.46%, which is considerably lower than in the previous experiment, and thus the importance of the chest sensor for correct discrimination between healthy and HD participants was demonstrated.

### C. Evaluation of the Linear Regression Model

The data from baseline simple MBT task for HD patients were used in a linear regression model of movement impairment in order generate an automatic MIS for new HD patients. The SVM regression algorithm with a linear kernel was used for this purpose [29]. Five most significant features from the baseline simple MBT classifier were used as independent variables for the regression model. Leave one out cross validation was used to evaluate the correlation between the automatically produced MIS and the mULMS of five chosen items related to chorea and dystonia.

**Fig. 6.**
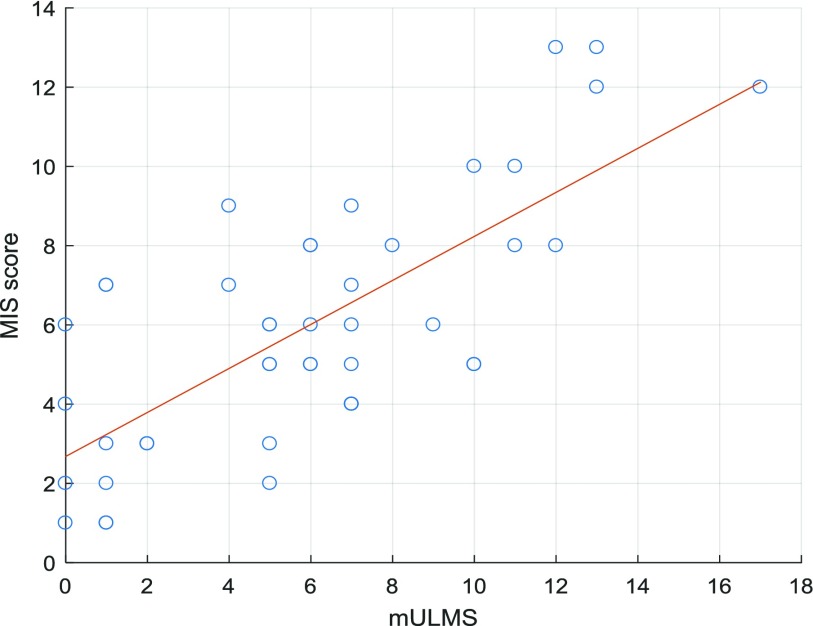
mULMS and corresponding automatic MIS generated by the linear regression model trained on all HD baseline simple task data apart from one of the samples used for testing.

The Pearson correlation coefficient $r $ between the automatic MIS and the mULMS was *0.77*, which corresponds to $r^{\textit {2}}= \textit {0.59}$ *and* $r^{\textit {2}}_{adj}= \textit {0.53}$ with a p-value < 0.01 indicating its high statistical significance. In addition, the mean absolute error (MAE) [31] between the automatic MIS and mULMS was *2.11*, which corresponds to the normalised MAE of *12.41*% with respect to the maximum score of *17* in the sample, thus showing an ability to predict the clinician rated upper limb chorea and dystonia scores with some accuracy. Fig. 6 shows the values of the mULMS and the corresponding automatic MIS generated by the linear regression model trained on all HD data apart from one of the samples used for testing.

## IV. Discussion

Here we present a system for an objective and continuous assessment of motor impairment during a novel upper limb task for HD patients. The system is based on data collected from tri-axial accelerometers, which were worn during the performance of a recently proposed MBT assessment of bilateral, upper motor function. Signal processing and machine learning methods were applied to the recorded accelerometer signals in order to produce an automatic MIS intended to reflect the degree of the movement impairment during the performance of the MBT.

A number of features, potentially useful for quantification of the movement impairment, were extracted from the accelerometer data and their significance in the discrimination task between healthy controls and HD participants was assessed. The results showed that temporal features were more important than frequency features, and in particular, features related to the non-dominant hand were just as significant if not more significant than those extracted from the data related to the dominant hand.

Before proceeding to the stage of generating an automatic MIS for HD patients, the extracted features were tested in a simpler discriminative task of differentiating between HD patients and healthy controls, in which very encouraging results were achieved, further proving that the extracted features captured the information relevant to motion impairment of HD patients. In this stage, three classifiers, one for each MBT task, were trained and tested, with the complex baseline classifier producing the best performance among the three classifiers. The results produced by the ensemble classifier demonstrate the advantage of using three tasks of increasing difficulty when performing MBT, with the complex baseline and dual task classifiers improving the accuracy of the baseline classifier when their results were combined. Given these results, we do not believe that the difference in ages between the HD and the healthy control groups had a detrimental effect on the ability of the assembly classifier to determine the group correctly on the basis of the accelerometer data, however will confirm this in subsequent studies. Furthermore, the importance of the chest sensor was assessed by removing the features related to this sensor from the dataset and repeating the above experiments and cross-validation using the reduced dataset. The results demonstrated significant increase in misclassifications indicating that the chest sensor is important in the assessment of HD movement impairment.

The system also produces a continuous value movement impairment score (MIS) that is well correlated with the clinician rated mULMS reflecting upper body chorea and dystonia. From a clinical perspective this is an exciting development in that the novel MIS provides a quantitative representation of chorea and dystonia of the upper limb in HD that traditionally is very difficult to rate reliably. The correlation between the automatic MIS and the mULMS demonstrates the viability of using the MIS for monitoring the progression of the movement disorders.

Nonetheless, further validation of the proposed system is required. Special care will need to be taken when comparing the automatic MIS and mULMS due to the subjectivity of the latter. There is a number of strategies which can be followed to address this issue, including testing intra- and inter-rater reliability.

## V. Conclusions

The approach presented in this study demonstrates the possibility of objective, consistent and sensitive assessment of the HD movement impairment using the MBT and three low-cost tri-axial accelerometers. The initial application of this test has been in HD: a highly characterised, single gene neurodegenerative disease. Given these promising results, we are now working to establish proof of concept in other neurological conditions such as Parkinson’s disease, tremor and dystonia. Future research will focus on combining acceleration with orientation data to improve the performance of the system and to expand the number of neurological movement disorders the system could assess.
